# Sound-encoded faces activate the left fusiform face area in the early blind

**DOI:** 10.1371/journal.pone.0286512

**Published:** 2023-11-22

**Authors:** Paula L. Plaza, Laurent Renier, Stephanie Rosemann, Anne G. De Volder, Josef P. Rauschecker

**Affiliations:** 1 Laboratory of Integrative Neuroscience and Cognition, Department of Neuroscience, Georgetown University Medical Center, Washington, DC, United States of America; 2 Neural Rehabilitation Laboratory, Institute of Neuroscience, Université Catholique de Louvain, Brussels, Belgium; Harvard Medical School, UNITED STATES

## Abstract

Face perception in humans and nonhuman primates is accomplished by a patchwork of specialized cortical regions. How these regions develop has remained controversial. In sighted individuals, facial information is primarily conveyed via the visual modality. Early blind individuals, on the other hand, can recognize shapes using auditory and tactile cues. Here we demonstrate that such individuals can learn to distinguish faces from houses and other shapes by using a sensory substitution device (SSD) presenting schematic faces as sound-encoded stimuli in the auditory modality. Using functional MRI, we then asked whether a face-selective brain region like the fusiform face area (FFA) shows selectivity for faces in the same subjects, and indeed, we found evidence for preferential activation of the left FFA by sound-encoded faces. These results imply that FFA development does not depend on experience with visual faces per se but may instead depend on exposure to the geometry of facial configurations.

## Introduction

Face perception is one of the most studied human skills because of its evolutionary relevance for survival [1–4]. Comparative evidence from primates and other mammals [5, 6] has shown the existence of face-selective neurons. Electrophysiological and neuroimaging studies in humans and monkeys have identified a distributed cortical and limbic network including frontal, temporal (STS), and occipital (OFA) regions which are specialized for processing facial information [7–14]. In humans, the most studied subregion of this specialized network is the fusiform face area (FFA), which is located in the fusiform gyrus within ventral temporal cortex. The FFA is highly selective for images of faces and face-like stimuli in comparison to images of scrambled faces or of houses, objects, bodies, and places [10, 11, 15–17]. In addition, the FFA plays a dynamic computational role in the detection, identification, and classification of faces [18] and is involved in many aspects of face perception, including both featural and configural (parts and whole) processing [19]. Some studies have reported hemispheric differences between left and right FFA, with the left FFA being more selective for the components (or parts) of a face and the right FFA more for the face as a whole [20–23]. The location of the FFA in the fusiform gyrus shows relatively little variation across adults, which is thought to be due, in part, to the correspondence between the morphology (cytoarchitecture and connectivity) of the fusiform gyrus and its computational function [24, 25]. This has further been taken to imply that the FFA and its selectivity for faces develop without visual experience [26, 27].

On the other hand, selectivity for face stimuli within the FFA develops gradually and becomes more finely tuned over the first decade of human development [28–36], suggesting that fusiform gyrus organization also depends on experience and learning. In fact, recent functional magnetic resonance imaging (fMRI) studies on the development of face selectivity in nonhuman primates (NHP) have demonstrated that “face patches” (cortical subregions with a high selectivity for faces) do not develop in monkeys deprived of vision [37, 38].

As facial information is predominantly conveyed through the visual modality in sighted humans, conveying facial information through another modality, such as hearing or touch in early blind (EB) individuals, could help to clarify the role experience plays in the specialization of the FFA. Visually deprived individuals thus offer a unique window into assessing how the morphological and functional architecture of the human brain develops and what role sensory experience plays in shaping the functional organization of the human brain.

**Fig 1:**
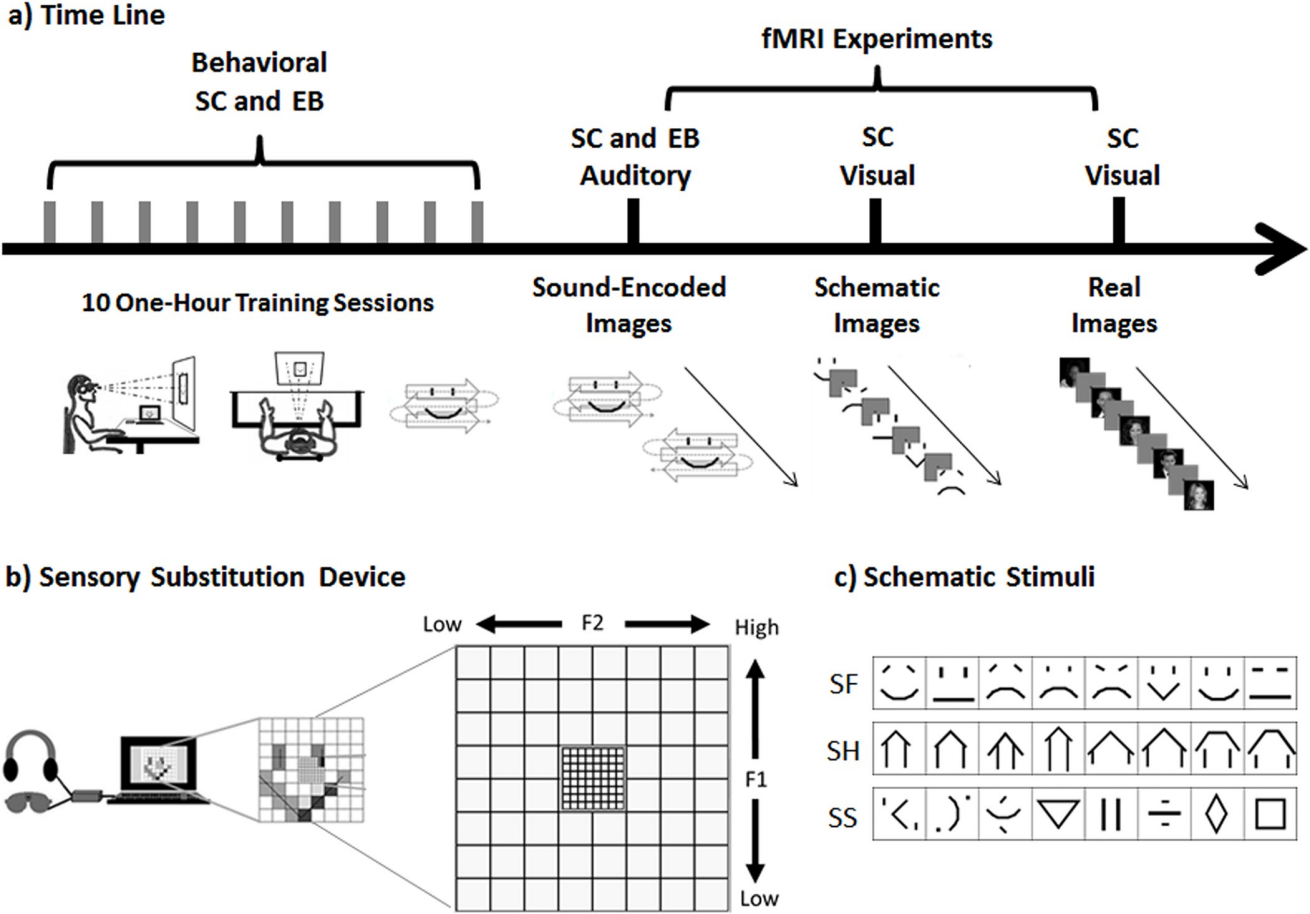
Experimental design. (a) Timeline illustrating the different steps in the study. Both EB (early blind) and SC (sighted control) subjects took part in behavioral tasks with the Sensory Substitution Device (SSD) and in fMRI tasks with images that were sound-encoded by means of the SSD. In a subsequent step, the 10 SC took part in visual tasks with schematic and real images. (b) The PSVA SSD and its ‘artificial retina’, illustrating a schematic face being perceived. (c) Schematic stimuli used in both SSD and visual schematic fMRI one-back comparison task. Three stimulus categories were tested: schematic faces (SF); schematic houses (SH); and schematic shapes (SS).

Sensory substitution devices (SSD) [39, 40] provide a means by which visual shape information can be conveyed to blind people via a preserved modality, such as audition or touch. One type of SSD converts two-dimensional visual images viewed through a head-mounted camera into audible signals modulated by scanning motions of the head [41–43], (see Material and Methods and Fig 1**)**. Previous functional neuroimaging studies have shown activation in various extrastriate areas of the visual cortex with similar devices in blind and sighted individuals [e.g. 42, 44–48]. In EB individuals, specialized areas of extra-striate cortex, such as the visual word form area (VWFA) and the extra-striate body area (EBA), respond selectively to sound-encoded stimuli presented via SSD [47, 48]. Based on such studies, it has been suggested that cortical regions selective for letter strings and body parts develop in the absence of visual input. However, despite the importance of the FFA for seeing faces, the extent to which this region is recruited by face stimuli presented via SSD in congenitally blind adults has never been tested. Only preliminary data have been presented by two studies in abstract form [49, 50].

In the present study, we investigated the effects of early blindness on the functional organization of the face perception network (including the FFA in both hemispheres) by using an SSD that was previously used successfully to study cross-modal spatial plasticity in the visual-auditory dorsal pathway [42, 43]. In particular, we asked to what extent the visual cortex of EB subjects is recruited to process sound-encoded schematic faces, in contrast to sound-encoded houses and other geometrical shapes.

We trained both sighted and blind subjects to process sound-encoded schematic stimuli that were transformed from the visual to the auditory modality via SSD. Using fMRI, we then examined activation of the fusiform gyrus, including the FFA in both hemispheres, by sound-encoded faces in EB individuals and sighted controls. If face-specific activation were found in the FFA of EB subjects trained and tested with sound-encoded faces, it would support the notion that a specific cortical region performs the same computational operation on the incoming input regardless of its sensory modality [43, 51]. In other words, “visual” cortex in the fusiform gyrus of blind individuals, as in sighted, has the capacity to develop into functionally specialized cortical modules, including for the processing of faces [52–55]. An alternative view of the role of the FFA is that its face specificity is really a predilection for highly similar stimuli that are difficult to distinguish from each other [56]. The aim of the present study was to contribute to this much-debated question as well.

## Material and methods

### Participants

We tested six EB participants (see Table 1) and ten sighted controls (SC, 4 females; 1 left-handed male; mean age = 27 yrs; SD = 6.2 yrs; age range = 20 yrs– 36 yrs), who were all trained to use a visual-to-auditory sensory substitution device (PSVA; 41) in the Laboratory of Integrative Neuroscience and Cognition at Georgetown University Medical Center, Washington, DC. Given the relative rarity of complete early blindness and the difficulty to recruit volunteers for several training sessions as well as fMRI testing, only six blind volunteers could be recruited and successfully completed all parts of the study. Aside from vision loss in EB, all volunteers were healthy with no history of neurological, psychiatric, or hearing problems. None of the volunteers had been trained to use an SSD prior to taking part in this study. All EB subjects were completely blind as a result of pregeniculate or ocular lesions that occurred before the age of two, and they did not recall having any visual experience. The study protocol was approved by the Institutional Review Board (IRB) of Georgetown University, according to the guidelines of the U.S. National Institutes of Health. Written informed consent was obtained from all participants prior to the experimental tasks. The participant shown in the Supplementary Video S1 File (**S1 File**; https://osf.io/ta8xw/) consented to having their image published under the Creative Commons Attribution license and signed the PLOS Consent Form for Publication in a PLOS Journal.

**Table 1:** Early blind subjects characteristics.

EB	Gender	Age	Cause of Blindness	Onset	Handedness
1	F	57	ROP-CB	Birth	Left
2	F	58	ROP-EB	< 2 years old	Right
3	M	40	Detached retina-EB	< 2 years old	Right
4	F	60	ROP-CB	Birth	Ambi
5	F	37	ROP-CB	Birth	Right
6	F	38	Glaucoma-EB	< 2 years old	Right

ROP: retinopathy of prematurity, CB: congenitally blind, EB: early blind, F: female, M: male

#### Experimental design

The study was divided into two steps for both EB and SC subjects: 1) learning and training to use the SSD, which included familiarization with the fMRI experimental set-up and SSD stimuli, and 2) fMRI data acquisition. In ten one-hour behavioral sessions, both EB and SC subjects were trained to identify two-dimensional (visual) patterns using the SSD with a head-worn camera. All subjects were informed about the category labels of the stimuli they were being trained on. SC subjects were blindfolded during the training sessions. During the last two sessions, both EB and SC subjects underwent familiarization with the fMRI testing conditions. They learned how to discriminate between different faces, houses and geometrical shapes by exploration with self-managed movements (learning phase) and then by hearing prerecorded sounds of the SSD that resulted from systematically scanning a given image in a standardized way, i.e., from an exploration pattern performed by the experimenter (testing phase). Thus, during the SSD-fMRI sessions, the brain activity of EB and SC subjects was measured while they listened to these prerecorded sounds (“soundscapes”) and attempted to perform the one-back task with their head fixed. The fMRI sessions consisted of three runs of the one-back task comprising the three stimulus categories: faces, houses and shapes. Stimulus onset was synchronized with the acquisition of the first slice of the epoch by the MRI scanner.

After having mastered the SSD conditions, SC subjects underwent two additional fMRI scanning sessions involving visual stimuli: one session included a visual version of the schematic stimuli used in the SSD conditions to make sure brain activation could be elicited in the Fusiform Face Area (FFA) using these simple visual stimuli. This control condition also assured a direct comparison of the effect of sensory modality in SC, i.e., auditory (via SSD) vs. visual. A second control session in SC involved photographs of actual faces, houses and geometrical shapes and served as the functional localizer to identify the regions of interest (ROIs) for the FFA in SC (Fig 1A). All SC subjects underwent testing with the visual conditions *after* having undergone all acquisitions with the SSD, so that they could not have been influenced (primed) by any memory of the visual version of the stimuli.

### Sensory substitution device (SSD)

#### Design and functional principles

The SSD used in the present study is referred to as a PSVA (prosthesis substituting vision with audition), [41]. The device consists of a video camera and headphones, connected to a computer that converts visual images into audible sounds in real time. The camera, which represents the artificial eye of the device, is attached to a pair of opaque lenses, which enables the sighted SSD-users to be blindfolded. The head-mounted camera provides an active-vision-like experience when exploring objects with the SSD, since its use involves similar sensory-motor contingencies as found in normal vision, i.e. head movements and associated sensory changes [57, 58]. The camera’s field of view comprises 64 pixels (low resolution), with the central zone having a resolution four times greater (i.e., pixels four times smaller) than the peripheral one. Each pixel of the camera’s field of view is associated with a single sound frequency, increasing from bottom to top and from left to right, generating continuous pitch changes during head movements along the vertical and horizontal axes. The horizontal axis of the artificial retina is related to stereophonic sound using binaural information from interaural intensity differences. Thus, if an image is presented in the left part of the visual field, the SSD-user perceives sounds coming from azimuth positions on the left, and vice versa for the right (Fig 1B). For instance, if the image is just a dot located in the superior right corner of the field of view of the camera, the related sound will be of high frequency and delivered mainly through the right headphone. If the dot is located in the top middle of the field of view, the sound will be a high frequency tone, but delivered through the right and left headphones at equal volume. If the image is a line at the bottom left corner, the associated sound will be a mixture of low frequencies delivered mainly through the left headphone. In order to decode more complex images than lines and dots, a greater amount of training involving active exploration behavior is needed.

#### Training to use the SSD

Subjects were trained over ten one-hour sessions to identify images encoded into sound patterns using the PSVA. While wearing the SSD, they sat in front of a board on which the images were displayed. At the beginning of training, subjects were asked to explore the image by moving their head back and forth in the horizontal plane (Fig 1). The complexity of the displayed images increased throughout the training.

At the end of training, two standardized, systematic ways of exploring the images were imposed: going from the top left corner and progressing horizontally line after line to the bottom right corner of the stimulus, and then going from the top right corner and progressing horizontally line after line to the bottom left corner of the stimulus (Fig 1B). The sound sequences corresponding to these two standard exploration patterns were recorded and later used during the familiarization sessions and the fMRI data acquisition. This procedure was developed for use in the MRI scanner, so that subjects could utilize the SSD without any motor actions (i.e., without the self-generated exploratory movements normally employed during SSD use). Thus, two standard prerecorded SSD sounds per stimulus existed for all fMRI studies. Subjects had to determine in a one-back comparison task whether each stimulus was the same as the previous one. Since two exploration variants were used for each stimulus, the same visual image had two different sound sequences or soundscapes associated with it, which forced subjects to actually decode the sounds rendered by the SSD to build a mental representation of the image and to perform the comparison task. At the end of the last training session, all subjects were able to correctly identify all stimuli with an accuracy of > 85% in all trials and no group difference was observed.

#### Auditory SSD-fMRI (schematic faces, houses and shapes encoded into sounds)

Each trial (15 s) contained a pair of stimuli from a single *category* encoded into sounds (e.g., two faces of 7 s each, separated by 1 s of silence). Face, house, and shape trials were interleaved in random order. The three runs comprised 18 trials each with equal probability of the *conditions* being presented. Subjects were required to determine whether the two stimuli were the same or different (one-back comparison task) by pressing a two-button response pad.

The one-back task involved three stimulus *categories*: faces, houses, and shapes. Each category then contained different *conditions* to be compared within the same category. For faces, there were sad, happy and neutral faces (modifying eyes and mouth); for houses, there were normal, wide, and tall houses (modifying roof and walls); and among the shapes there were triangles, squares, and rectangles. Therefore, the difficulty of the task in the three categories was similar, it involved a one-back task, where subjects had to decide whether the second image encoded in the sound domain corresponded to the same or a different condition within that category. The following examples may serve to illustrate the behavioral task: If a happy face was presented and then a sad face, the subject had to respond that they were “different”. If two happy faces were presented, then the answer would be "same". If a square and a triangle were presented, the answer was “different”, if a tall house and a wide house were presented, the answer was “different”, if both houses were high, the answer was “same”.

It is also worth noting that we systematically used two versions of the rendered sounds resulting from the standard explorations (i.e., created by two different exploration patterns; top-left-to-bottom-right and top-right-to-bottom-left; Fig 1A) when two of the same stimuli followed each other, so that subjects had to base their judgment on the original visual representation of the stimuli rather than on simple acoustic matching.

#### Visual fMRI sessions (schematic and real objects)

These conditions used a visual representation of the schematic stimuli used for the sound-encoded stimuli or photographs of real faces, geometrical shapes or houses (see Fig 1C). Stimuli were presented in a block design (faces, houses, shapes), with the order of the blocks randomized across the four fMRI runs, two for the schematic stimuli and two for the real “objects”. There were 9 blocks in each run. In each block, 48 images were presented for a duration of 600 ms. Each block was followed by a 12-s rest period. A fixation point was displayed (500 ms) at the center of the screen during inter-stimulus intervals and resting periods. All SC subjects were instructed to gaze at the fixation point, in order to limit head and eye movements during scanning. Subjects were required to press a button when a stimulus was repeated.

#### MRI data acquisition

MRI data were acquired at the Center for Functional and Molecular Imaging at Georgetown University using a 3-Tesla Siemens Tim Trio scanner with an echo-planar imaging (EPI) sequence on a 12-channel head coil (flip angle = 90°, TR = 3004 ms, TE = 30 ms). The field of view (FOV) was 220 x 220 mm^2^ with 64 x 64 matrix size; slice thickness was 2.3 x 2.3 x 2.3 mm^3^. Each volume of 51 slices was acquired using continuous sampling in an interleaved fashion.

A 3D multiband gradient echo-planar imaging (EPI) sequence with a 64-channel head coil was used to acquire the fMRI data for the visual schematic task. Scanning parameters included 69 transverse slices with phase encoding in the A>>P direction; 0.54 ms echo spacing, fat saturation; flip angle = 30°, TR = 3000 ms; TE = 34 ms; FOV = 200 x 200 mm^2^; slice thickness = 2 x 2 x 2 mm^3^; Acceleration factor slice = 3. The parameters for the T1-weighted structural MPRAGE were 176 sagittal slices in the A>>P direction, flip angle = 9°, TR = 1900 ms, TE = 2.52 ms, 1 x 1 x 1 mm^3^ voxel resolution.

#### Data analysis

Data analyses were performed using the BrainVoyager QX software package (Version 2.8, BrainInnovation) and SPM12 (www.fil.ion.ucl.ac.uk), MATLAB R2016a (Mathworks, Nautik, MA). Functional MRI data were preprocessed with slice scan timing correction, temporal high-pass filtering (removing frequencies lower than 3 cycles/run) and head motion correction. For anatomical reference, the computed statistical maps were overlaid on the 3-D T1-weighted scans. Therefore, functional and anatomical data were co-registered and transformed into Talairach space. In each individual, the predictor time courses of the regression model were computed on the basis of a general linear model of the relationship between neural activity and hemodynamic response function (HRF), assuming an HRF neural response during phases of active conditions.

#### Whole-brain and region-of-interest (ROI) analyses

**Fig 2:**
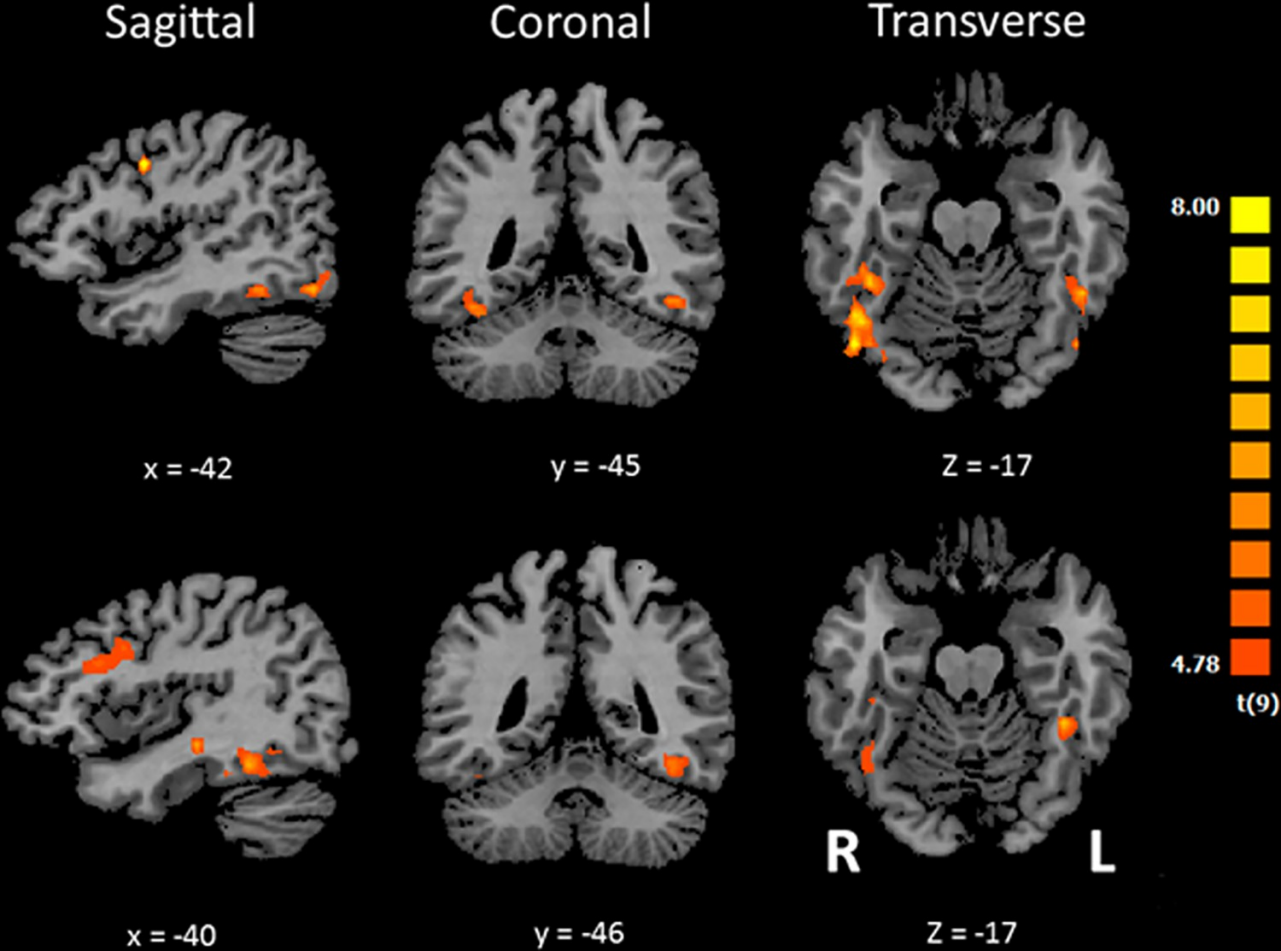
Visual activation of the fusiform face area (FFA) in SC subjects viewing schematic faces. The upper part of the Fig shows the activation maps obtained using the contrast [Schematic Faces minus Rest] in 10 subjects. The lower part of the Fig shows the activation maps obtained using the contrast [Schematic Faces minus Schematic Houses]. Activation maps were obtained using a threshold of qFDR < 0.05 in combination with a cluster size threshold correction of p < 0.01. Brain activation foci (positive values only) were superimposed on sagittal, coronal and transverse views of the normalized MRI brain of a representative subject.

**Fig 3:**
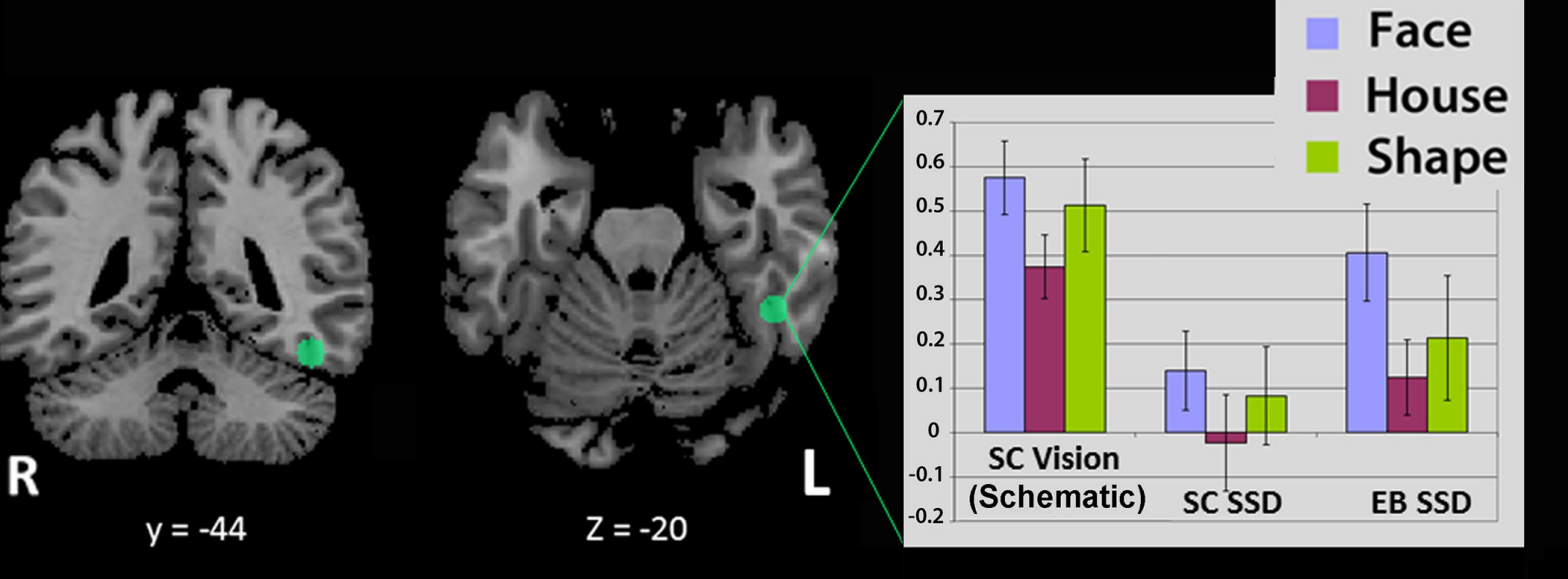
Brain activity within the Fusiform Face Area Region of Interest (FFA-ROI) in early blind (EB) and sighted control (SC) subjects.

Data from the visual conditions in SC subjects were analyzed using a random-effects (RFX) approach at the whole-brain level to identify the brain areas involved in the visual processing of faces, houses and geometrical shapes. Results of these analyses were used to identify and circumscribe the ROIs that were further used in ROI analyses in the SSD conditions. The brain activation foci that were localized in the closest vicinity of the FFA according to coordinates reported in the literature were examined and 5-mm radius spheres (515 voxels) were created around the main voxel peaks located within these significant clusters found in the functional (visual) localizer. The following ROI-sphere was obtained for the FFA (see green filled circle in Fig 3**)**: left FFA, x: -44; y: -44; z: -18. Raw beta values were then extracted from this spherical region for each condition (schematic Faces, Houses and Shapes) and group (SC Vision, SC SSD, EB SSD). These beta values were plotted in bar graphs, as shown in Fig 2. Individual Student t-tests were performed to obtain relevant comparisons. In addition, nonparametric testing was performed to compare the beta values of the relevant comparisons: a Mann-Whitney U test was used for between-group (EB versus SC) comparisons and a Wilcoxon signed rank test was used to compare beta values for each condition within the EB.

#### Single-subject analyses

Individual data of the PSVA task were pre-processed using SPM12 (www.fil.ion.ucl.ac.uk) and MATLAB R2016a (Mathworks, Nautik, Massachusetts). Slice timing correction was conducted using the slice collected at the halfway point as a reference slice (slice 51 for PSVA EPI sequence and 1555 ms for the multiband EPI sequence). Slice-timing-corrected images were realigned to the first image using a least-squares approach and a 6-parameter (rigid-body) spatial transformation and were then co-registered to a participant’s MPRAGE using 4^th^ degree B-Spline interpolation. Data were then spatially normalized to an MNI template using 4^th^-degree B-Spline interpolation and underwent 3-dimensional spatial smoothing of 4-mm FWHM (isotropic Gaussian kernel). In the single-subject analyses, preprocessed data were analyzed using a fixed-effects general linear model (GLM). Regressors modelling each stimulus condition were convolved with the canonical hemodynamic response function implemented in SPM12. Head motion parameters were included as regressors of no interest. Time and dispersion derivatives were also modeled and the resulting SPM *F*-maps for the different contrasts for each individual subject were masked with a BA37 mask defined by the AAL atlas and created using MATLAB R2016a. ROI analyses involved identifying significant clusters of activation corresponding to the location of the FFA. Results are displayed with a corrected threshold of q-FDR < 0.05 (with p < 0.01 cluster-forming threshold).

## Results

### Behavioral training and testing

Six early blind (EB) and ten sighted controls (SC) were trained to identify sound-encoded schematic images of faces, houses, and geometrical shapes using a visual-to-auditory sensory substitution device (SSD) referred to as the PSVA (Fig 1A and 1B; [41]). Both EB and SC participants were able to correctly identify all stimuli with greater than 85% accuracy by the end of their training. Following training, each participant completed three runs of a one-back comparison task inside the MRI scanner; stimuli consisted of sound-encoded schematic images of faces, houses, and geometrical shapes with similar complexity (Fig 1C). The visual-to-auditory SSD transformation of the schematic images was conducted in a standardized manner in two different scanning movement directions (Fig 1A), such that the task could not simply be completed based on acoustic template comparison but required shape reconstruction of the sound-encoded image and its identification (see Material and Methods and Supplementary Video S1 File [https://osf.io/ta8xw/] for details).

In a further fMRI session (Fig 1A, right), the ten SC subjects were presented with the original visual versions of the schematic stimuli used in the SSD task. In addition, photographic images of real objects (i.e., faces, houses and geometrical shapes) were presented in separate fMRI runs using a one-back comparison task. The sighted group scored an average of 90% on the visual one-back task inside the scanner.

### Activation within the FFA

Six EB and ten SC participants completed the one-back PSVA task which comprised sound-encoded images of the schematic stimuli that were also used as one type of visual stimuli in the SC (Fig 1C). Given our a-priori hypothesis about the brain structure where effects were expected, we first performed a region-of-interest (ROI) analysis based on the FFA region, as identified by a visual localizer performed in an independent data set of sighted participants observing pictures and schematic visual images (see Material and Methods).

#### Visual task and functional localizer

Ten sighted controls took part in the visual one-back comparison task. The visual task incorporated photographs of real faces, houses or geometrical shapes as well as the visual version of the sound-encoded schematic stimuli (drawings) used in the PSVA task. These conditions were used as localizers for identifying the FFA region (Fig 1C).

Whole-brain random effects (RFX) analyses using a corrected threshold of q-FDR < 0.05 (with p < 0.01 cluster-forming threshold) were performed in SC subjects. The contrast [Schematic Faces minus Rest] showed predominantly right-sided activation of the fusiform gyrus (see upper part of Fig 2). The same contrast using photographs of real faces, [Photographic Faces minus Rest], revealed a similar but stronger and more extended bilateral activation in the fusiform gyrus (see S1 Fig). The contrast [Schematic Faces minus Schematic Houses] showed a focus of activation only in the left fusiform gyrus (see lower part of Fig 2). This activation focus largely overlapped with the focus obtained in the left fusiform gyrus (x: -39; y: -41; z: -19; 284 voxels) using the contrast [Photographic Faces minus Photographic Houses], with [Photographic Faces minus Rest] as an inclusive mask (see S1 Fig and S2 Table). The coordinates of the activation focus were in the vicinity of the region referenced as the left FFA in the literature [11, 20, 56, 59, 60]. No other brain activation focus was found in the left or right fusiform gyrus at the selected threshold (p<0.05, uncorrected; S1 Table). Therefore, we decided to focus on the left FFA ROI.

### Region-of-interest (ROI) analysis

**Fig 4:**
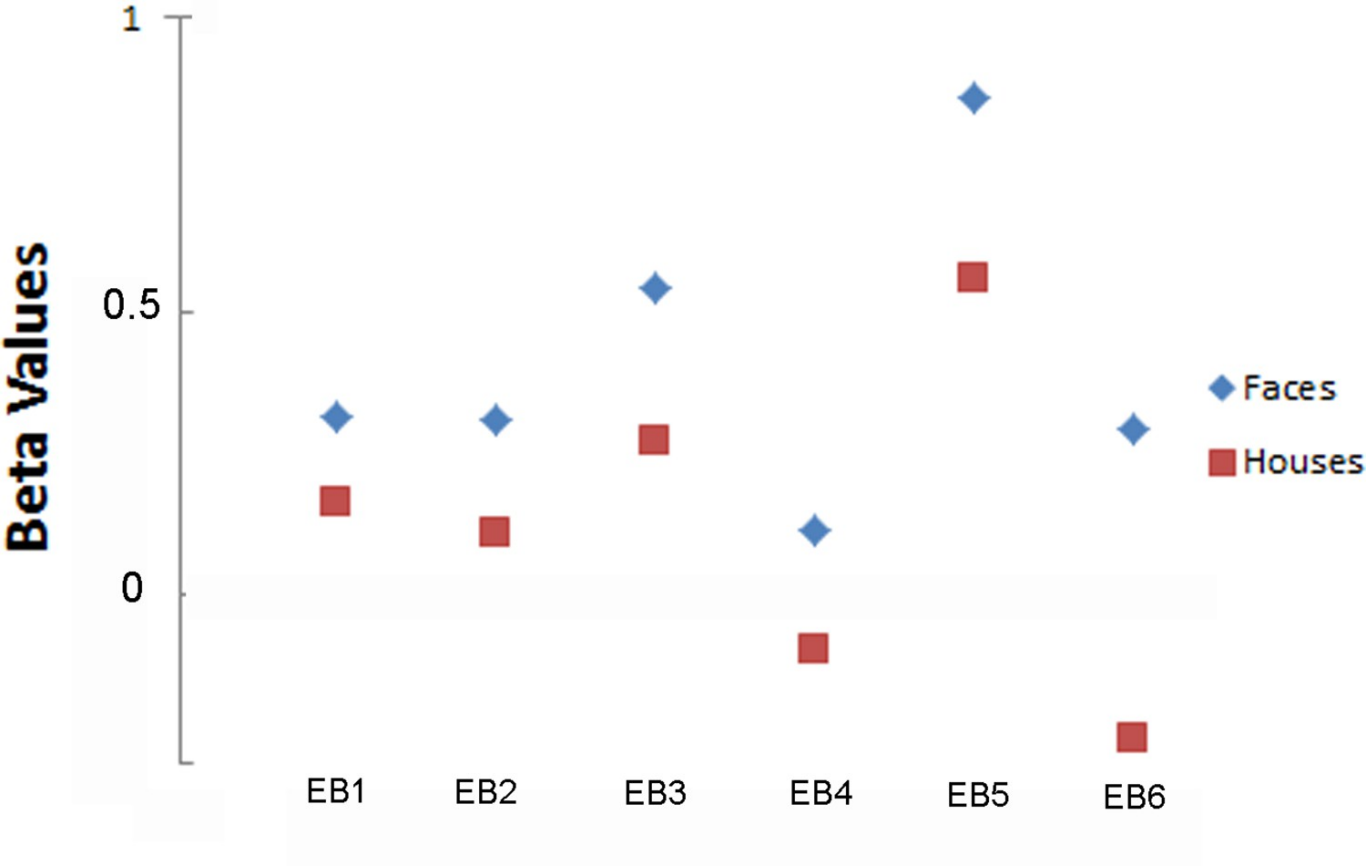
Individual beta values for responses to SSD faces and houses within the left FFA-ROI. Scatter plots of the single-subject (raw) beta values are displayed for the EB group.

A 5-mm radius sphere (515 voxels, see green filled circles in Fig 3) was created around the main voxel peak (x: -44; y: -44; z: -18) located within the significant cluster found in the functional (visual) localizer using schematic stimuli in the sighted group (see lower part of Fig 2). Unlike real photographic faces, which regularly elicit a selective response in the FFA bilaterally (often predominantly on the right; S1 Fig), schematic faces elicited a focal response predominantly in the left FFA (see Material and Methods). ROI analyses across groups revealed a significant response to sound-encoded schematic faces in the EB group (EB SSD) within the left FFA (see Fig 3). Student t and Wilcoxon signed-rank tests both confirmed that the hemodynamic response for face stimuli was significantly greater than for the house condition (faces > houses; t = 3.719, p = 0.014; W = 21, p = 0.031). All six EB subjects showed brain activity in the FFA that was higher than the activation by schematic houses during the SSD-Face conditions (see scatter plot of beta values in Fig 4).

A 5-mm radius sphere (515 voxels) was created around the main voxel peak (x: -44; y: -44; z: -18) located within the FFA, as identified by a functional (visual) localizer in the SC, using the contrast [Schematic Faces minus Schematic Houses] (see Fig 2, lower part).

The bar graphs on the right show the BOLD response (raw beta value) in the spherical FFA-ROI as a function of group (SC vision, SC SSD, and EB SSD) and condition/stimulus (Face, House, and Geometrical Shape). Beta values for the schematic visual conditions in SC are provided as a reference on the left. In EB subjects, the Face condition was significantly above the baseline (t = 3.72, p<0.02) and above the House condition (t = 4.85, p < 0.005). See S1 Table for detailed statistical results of the comparisons. Error bars represent standard error of the mean. L: left; SSD: sensory substitution device; SC: sighted controls; EB: early blind.

### Whole-brain analyses

To visualize activation patterns and their specificity, whole-brain analyses were also performed. The contrast [Schematic Faces minus Schematic Houses] in the six EB subjects showed an activation focus in the left FFA (135 voxels, see S1 Table**, penultimate line, in bold**). In SC, the same contrast, [Schematic Faces minus Schematic Houses], did not show a significant activation focus in the fusiform gyrus at the selected threshold.

## Discussion

In the present study, we demonstrate that perception of schematic faces with a visual-to-auditory sensory substitution device (SSD, PSVA) activates the fusiform gyrus (BA 37) in EB subjects, in a region identified as the left fusiform face area (FFA) in sighted subjects. The fact that the FFA is activated in the blind by sound-encoded patterns of faces suggests that the computational algorithms for the detection of faces can develop without experience in the visual domain. Training and testing with the SSD and subsequent brain imaging reveals the presence of face-specific modules that seem to resonate with the underlying geometry of the recipient structure.

Our results are the first to document that a specific, prominent region of the ventral pathway, the FFA of the left cerebral hemisphere, shows a preference for the perception of sound-encoded schematic faces in EB subjects. Activation of the FFA observed in sighted individuals when they perceived the same schematic faces visually, corresponded to the same location that had previously been found to identify that cortical region [61].

### Role of the left FFA in component rather than configural face perception

In the current study, only the left FFA in the EB group showed a preference for sound-encoded images of schematic faces. This is in line with previous evidence of left-hemispheric involvement in analytic processing of local facial parts or features, as opposed to configural or ‘holistic’ face perception, which engages mostly the right FFA, [62–64]. Interestingly, there is also evidence of holistic processing in the OFA [63–64], however, some studies have claimed a mix of holistic and part-based processing in the FFA [65–69]. Sergent [21, 22], in a series of classical studies, showed that left-hemisphere lateralization of face processing is more likely to occur for stimuli of high spatial frequency and when the tasks required component rather than configural analysis of the face (parts rather than whole). Similar conclusions came from two different labs specialized in face processing [20, 23], which seem to agree that the left FFA is involved in “early-sketch” component processing of faces, because it responds better to parts of faces than to their configuration. For the same reason, the left FFA may be favored by the sequential stimulation with an auditory SSD.

Although there is strong evidence for the analytical processing of schematic faces, there is also evidence in favor of schematic faces that show holistic processing [70], demonstrating that schematic faces are not processed sequentially per se. However, in the present study the analytic processing in EB subjects depends specifically on the use of an SSD. In other words, the stimulus perception using an SSD, such as the PSVA, is typically analytical and sequential, element by element, facilitating left recruitment, but maintaining specific recruitment of brain areas: face processing.

Another study [71] focused on the face inversion effect, defining the term relationally (at a level between featural and configural), as referring to the coding of spatial (or geometrical) relationships between facial components, which seems to be essential for face recognition. Consistent with our findings, line drawings of faces reduce configural processing and, therefore, the involvement of the right FFA. From these studies it becomes clear that the left and right FFA both contribute to face processing in their own way. The question remains whether both hemispheres do so independently or whether there is an asymmetric relationship between them. Connectivity studies might clarify the contributions of each hemisphere to the specific processing of faces through an SSD.

What is obvious is that the left FFA makes a substantial contribution to face processing, so the assumption is warranted that, being able to process relational information about faces, blind participants using an SSD should be able to gain some semblance of face recognition as in haptic face processing [72]. Whether this ability carries enough information to ultimately identify individual persons, remains to be seen in future studies. It is equally possible that blind individuals remain in an immature state, reflecting the lack of synaptic pruning during critical periods of early visual plasticity, as early blind people have been shown to perform worse on face-perception tasks because they rely more on featural and less on configural processing than do adults [34].

### Decoding of facial expressions

Some of our blind participants were clearly able to distinguish different emotions (‘happy’ or ‘sad’) in the various versions of schematic faces they were presented with (see Supplemental Video S1 File, https://osf.io/ta8xw/), although this was not systematically tested. EB individuals spontaneously produce facial expressions of basic emotions that are very similar to those produced by sighted individuals in the same situations [73]. This cannot be accomplished by imitation based on visual observation, which is unavailable to them [27, 72, 74, 75]. It has previously been shown that haptic identification of facial expressions in the EB activates the same regions in the inferior frontal and middle temporal gyri as in a sighted control group, with predominant activation in the left hemisphere, as in the present study [72]. Thus, the functional organization of the face processing network may be further enhanced through kinesthetic feedback from self-produced facial movements. Taken together, the current data suggest that internal models may exist as brain systems that couple the perception and production of facial expressions, similar to internal models in speech and language [58, 76].

#### Mechanisms of developmental plasticity in the face processing system

The central finding in the current study was that all EB participants consistently demonstrated higher activation by faces over houses in the left FFA. Taken together, these results support the notion that the FFA as a whole is predisposed towards detecting and identifying distinct face configurations [77] including those corresponding to basic facial expressions. Studies that include newborns [29, 78, 79] are needed to specify the neurocognitive development of face processing and the innate versus acquired aspects of FFA function [37, 38]. Nevertheless, as the present study clearly attests, individuals without visual experience activate the left FFA during perception of schematic faces with an SSD. This speaks to the fact that either nonvisual stimuli with a face-like configuration or geometry are sufficient for the development of face domains (particularly in the left hemisphere using schematic faces), or that at least some computational aspects of FFA function are morphologically pre-specified [24, 25]. Functional connectivity studies using the left FFA as a seed region might help to provide additional information about the functional role of the left FFA in EB subjects [26].

The present results agree with previous studies that have shown preserved functional organization in the extrastriate body area (EBA) [48] and the visual word form area (VWFA) [47] in EB individuals using different types of SSD [80–84]. These outcomes provide further support for the hypothesis that cortical areas commonly considered part of extrastriate cortex can develop or maintain their functional-computational specialization in the absence of visual input. Although extrastriate areas that appear visual in sighted adults may have more multisensory potential than previously thought, specific regions retain or develop the same functional role in the EB that they assume in sighted individuals. Thus, although the sensory modality of input fibers driving the neurons in reorganized occipital cortex of blind individuals may differ, the computational functions of extrastriate areas are stable, regardless of visual experience.

### General conclusions and limitations of the study

In the present study, EB individuals were able to recognize schematic faces using sound patterns generated by a sensory substitution device (SSD) that translates the spatial layout of visual stimuli into the auditory modality. Head movements of the blind participants scanning two-dimensional visual objects (faces, houses, and geometrical shapes) led to the perception of time-varying auditory shapes that were homologous in structure to the original visual shapes. Our finding that blind individuals without prior visual experience recruited a specialized ventral-stream region like the FFA for the perception and interpretation of specific sound patterns is significant for several reasons: It supports the notion that the ventral pathway contains modules for the perception of specific object categories and that its nonvisual functions are, therefore, not a ‘side-effect’ of mental imagery. Although top-down influences cannot be ruled out, the basic organization of the ventral stream is that of a hierarchical, bottom-up system with potentially supramodal character. Moreover, observing that the FFA was activated by face perception via a visual-to-auditory SSD demonstrates that the blind visual cortex has the ability to develop much of its functional organization in the absence of visual experience. Non-visual experience can replace the visual input in its important function of driving these computational modules during development, as long as it resonates with the structural requirements of the recipient network.

### Role of expertise in defining the FFA

Faces are known to be similar stimuli that share the same parts in the same organization [85]. Some accounts [86–88] have proposed, therefore, that the FFA is not a domain-specific module for the recognition of faces, but rather reflects expertise for object categories consisting of highly similar stimuli that one has learned to classify, e.g., birds for bird experts, or cars for car experts (and faces for most sighted people, which can be considered face experts.) Extensive training with sound-encoded faces in the blind might create expertise for the corresponding sounds (provided that responses get shifted from the visual to the auditory modality by cross-modal plasticity). Thus, the blind person might label all sounds from that category a ‘face’ because that is what he or she has been told (as opposed to a house or a shape in the other two categories). In this view, the right FFA deals with whole faces (or other expertise-related objects) on a configural basis, whereas the left FFA deals with their parts or specific spatial components [20]. It is worth noting that the training of the volunteers prior to our fMRI study was performed with stimuli from all three categories. This should rule out any training-related differences as an explanation for the brain activation patterns between stimulus categories.

It is likely, therefore, that the obvious propensity of the FFA towards faces is not completely random but relates to built-in mechanisms and predispositions discussed earlier. In that context, it is interesting that the expertise effect in the FFA is found mainly or exclusively in the right hemisphere [56]. The left FFA, by contrast, shows no expertise effect. This makes it even more likely that the effects found in our blind participants are due to cross-modal plasticity in the domain of face perception.

It was also noted by some of the same authors that novices may use a featural strategy, whereas experts may use a more configural strategy [86–88]. How the different strategies relate to behavioral performance is an open question. Perhaps only configural processing is a good predictor of behavioral expertise. Whether this has consequences for the limits of SSD-trained blind persons’ abilities to ultimately recognize faces needs further exploration. As mentioned earlier, such persons resemble children with immature vision, who also rely more on featural and less on configural processing than do adults [34].

Nevertheless, one might reasonably ask whether the contrast categories (houses and geometrical shapes) of our experimental design had the same overall similarity as faces, or whether they were less similar. If the latter, then it might not be the faces per se that specifically elicit the effect but more generally a category whose stimuli, once sensory-substituted, were more similar to one another than the stimuli of the contrast categories. To address this concern, follow-up studies could be performed in a larger number of blind participants and sighted controls. In addition, future studies could counterbalance the design, so that the “face sounds” in one group of blind people become the “object sounds” in the other group. This would demonstrate that the left FFA effect arises from the “face sounds” per se (controlled for stimulus similarity as detailed above), not from the instructions that labelled some sounds as “face sounds.”

### Influence of age and gender

Finally, future studies with larger cohorts of participants should also consider matching blind and sighted participants for age and gender. Only a few studies have investigated gender differences in brain activation during the encoding and recognition of faces [89], and if they did, they only tested photographic faces. The same is true for the effects of aging: Only one study compared the effects of age on processing synthetic faces representing configural information similar to ours [90]. In that study, older adults failed to show adaptation to the same face repeatedly presented in the same view in the right FFA, which elicited the most adaptation in young adults. Age was not related to cortical thickness but was related to resting-state metabolic activity [91], suggesting a detuning of face-selective mechanisms in older adults [92]. On the other hand, young adults showed functional connectivity between the right FFA and its homologous region during face processing, whereas older adults did not engage the left FFA.

## Supporting information

S1 Fig Visual activation of the fusiform face area (FFA) in SC subjects viewing photographs of real faces. The Figure shows activation maps obtained in 10 sighted subjects using the contrast [Real Faces minus Rest]. These activation maps were used as inclusive masks in further analyses of the FFA. The maps were obtained using a corrected threshold of qFDR < 0.05 in combination with a cluster size threshold correction of p < 0.01. An activation focus was found in the left fusiform gyrus (x: -39; y: -41; z: -19, 284 voxels, see **S2 Table**), which we identified as the left FFA.
(TIF)

S1 File Video recording of a blind participant using the PSVA to explore a schematic face. Recognition of schematic visual patterns by a blind volunteer using a sensory substitution device (SSD). The SSD converts two-dimensional visual images viewed through a head-mounted camera into audible signals modulated by scanning motions of the head. (Video can be downloaded from https://osf.io/ta8xw/).
(MP4)

S1 Table Activation maps in EB subjects resulting from an RFX whole-brain analysis (p<0.05 (uncorrected)), using the contrast [Schematic Faces minus Schematic Houses] (see Fig 2, lower part). ParaHippo G: parahippocampal gyrus, Post CG: posterior cingulate gyrus, Med FG: median frontal gyrus, Mid FG: middle frontal gyrus, SFG, superior frontal gyrus, Prec G: precentral gyrus, IPL: inferior parietal lobule, FFA: fusiform face area, IFG: inferior frontal gyrus.
(DOCX)

S2 Table Visual activation of the fusiform face area (FFA), [photographic faces minus photographic houses] in inclusive mask [photographic faces vs rest] q(FDR) < 0.05 with cluster size threshold of p < 0.01, see S1 Fig. STG: superior temporal gyrus, Mid TG: middle temporal gyrus, SFG: superior frontal gyrus, Cing G: cingulate gyrus, ITG: inferior temporal gyrus, FFA: fusiform face area.
(DOCX)
