# Supplementary figures and images for: Sound-encoded faces activate the left fusiform face area in the early blind
Source: PLoS One. 2023 Nov 22;18(11):e0286512. doi: 10.1371/journal.pone.0286512 (PMC10664868; doi:10.1371/journal.pone.0286512)

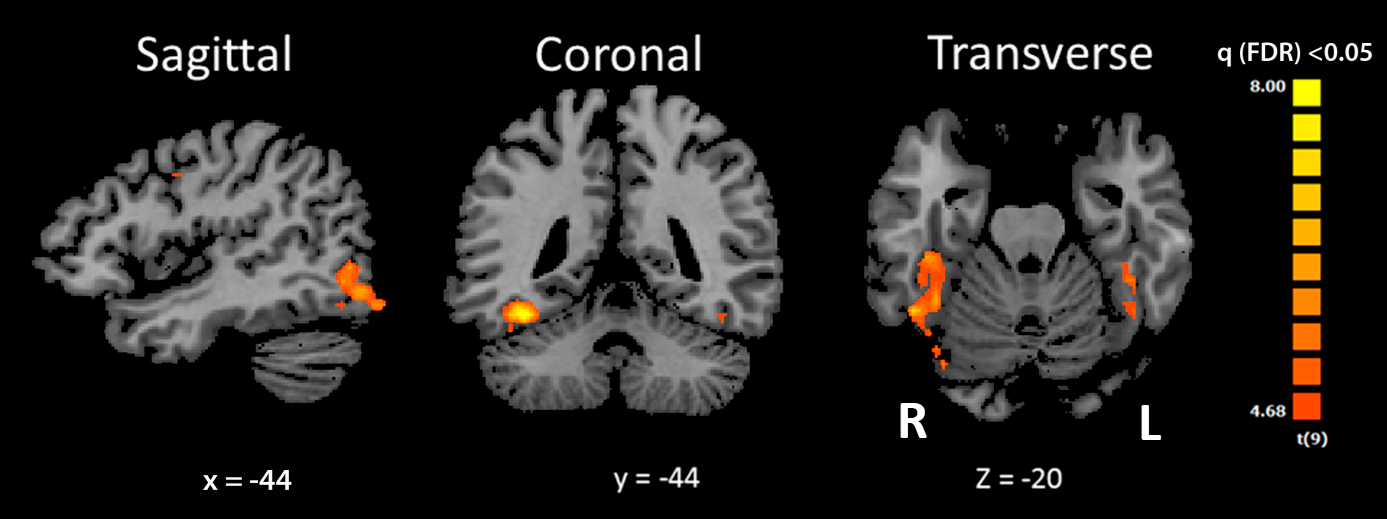

Supplement: S1 Fig — The Figure shows activation maps obtained in 10 sighted subjects using the contrast [Real Faces minus Rest]. These activation maps were used as inclusive masks in further analyses of the FFA. The maps were obtained using a corrected threshold of qFDR < 0.05 in combination with a cluster size threshold correction of p < 0.01. An activation focus was found in the left fusiform gyrus (x: -39; y: -41; z: -19, 284 voxels, see S2 Table), which we identified as the left FFA. (TIF) [file pone.0286512.s001.tif]
