# Supplementary material for: Sound-encoded faces activate the left fusiform face area in the early blind
Source: PLoS One. 2023 Nov 22;18(11):e0286512. doi: 10.1371/journal.pone.0286512 (PMC10664868; doi:10.1371/journal.pone.0286512)
Supplement: S1 Table — ParaHippo G: parahippocampal gyrus, Post CG: posterior cingulate gyrus, Med FG: median frontal gyrus, Mid FG: middle frontal gyrus, SFG, superior frontal gyrus, Prec G: precentral gyrus, IPL: inferior parietal lobule, FFA: fusiform face area, IFG: inferior frontal gyrus. (DOCX) [file pone.0286512.s003.docx]

**S1 Table.** Activation maps in EB subjects resulting from an RFX whole-brain analysis (p<0.05 (uncorrected)), using the contrast [Schematic Faces minus Schematic Houses] (see **Fig 2**, lower part**).**

| **Brain region** | **Brodmann area** | **Coordinates (x, y, z)** | | | **Cluster size** | **t value** | **p value** |
| --- | --- | --- | --- | --- | --- | --- | --- |
| Right ParaHippo G | BA 36 | 41 | -35 | -11 | 170 | 42.833 | 0.00784 |
| Right Post CG | BA 3 | 34 | -23 | 44 | 210 | 44.866 | 0.00648 |
| Left Precuneus | BA 31 | -1 | -61 | 25 | 265 | 61.454 | 0.00166 |
| Left Med FG | BA 6 | -19 | 29 | 35 | 432 | 52.364 | 0.00336 |
| Left Mid FG | BA 8 | -29 | 19 | 37 | 502 | 73.574 | 0.00144 |
| Left SFG | BA 10 | -30 | 49 | 14 | 382 | 56.808 | 0.00236 |
| Left Prec G | BA 6 | -31 | 5 | 27 | 180 | 48.308 | 0.00475 |
| Left IPL | BA 39 | -40 | -67 | 41 | 197 | 38.996 | 0.01141 |
| **Left FFA** | **BA 37** | **-43** | **-44** | **-14** | **135** | **47.157** | **0.00785** |
| Left IFG | BA 9 | -42 | 8 | 27 | 199 | 41.742 | 0.00870 |

ParaHippo G: parahippocampal gyrus, Post CG: posterior cingulate gyrus, Med FG: median frontal gyrus, Mid FG: middle frontal gyrus, SFG, superior frontal gyrus, Prec G: precentral gyrus, IPL: inferior parietal lobule, FFA: fusiform face area, IFG: inferior frontal gyrus
