# Supplementary material for: Sound-encoded faces activate the left fusiform face area in the early blind
Source: PLoS One. 2023 Nov 22;18(11):e0286512. doi: 10.1371/journal.pone.0286512 (PMC10664868; doi:10.1371/journal.pone.0286512)
Supplement: S2 Table — STG: superior temporal gyrus, Mid TG: middle temporal gyrus, SFG: superior frontal gyrus, Cing G: cingulate gyrus, ITG: inferior temporal gyrus, FFA: fusiform face area. (DOCX) [file pone.0286512.s004.docx]

**S2 Table.** Visual activation of the Fusiform Face Area (FFA), [Photographic Faces minus Photographic Houses] in inclusive mask [Photographic Faces vs Rest] q(FDR) < 0.05 with cluster size threshold of p < 0.01, see **S1 Fig**.

| **Brain region** | **Brodmann area** | **Coordinates** | | | **Cluster size** | **t value** | **p value** |
| --- | --- | --- | --- | --- | --- | --- | --- |
|  |  | **(x** | **y** | **z)** |  |  |  |
| Right Mid TG | BA 21 | 56 | -6 | -15 | 131 | 65.943 | 0.0001 |
| Right STG | BA 22 | 48 | -43 | 11 | 235 | 121.036 | 0.000001 |
| Right Globus Pallidus |  | 17 | -4 | -8 | 103 | 66.131 | 0.000098 |
| Right Cuneus | BA 23 | 8 | -71 | 11 | 394 | 93.913 | 0.000006 |
| Left SFG | BA 9 | -4 | 54 | 33 | 554 | 80.325 | 0.000021 |
| Left Cing G | BA 31 | 0 | -52 | 25 | 325 | 64.732 | 0.000115 |
| Left Ant Cing | BA 32 | -3 | 48 | -3 | 692 | 72.924 | 0.000046 |
| Left Cuneus | BA 18 | 0 | -72 | 19 | 231 | 73.087 | 0.000045 |
| Left Cing G | BA 24 | -1 | -15 | 36 | 194 | 103.937 | 0.000003 |
| Left Lingual G | BA 18 | -8 | -70 | 7 | 357 | 74.097 | 0.000041 |
| Left SFG | BA 8 | -20 | 40 | 44 | 149 | 56.829 | 0.000301 |
| **Left FFA** | **BA 37** | **-39** | **-41** | **-19** | **284** | **72.671** | **0.000047** |
| Left STG | BA 22 | -42 | -20 | -8 | 106 | 99.898 | 0.000004 |
| Left STG | BA 22 | -47 | -58 | 15 | 249 | 82.857 | 0.000017 |
| Left STG | BA 22 | -54 | -42 | 8 | 128 | 101.575 | 0.000003 |
| Left ITG | BA 21 | -58 | -8 | -14 | 373 | 87.457 | 0.000011 |

STG: superior temporal gyrus, Mid TG: middle temporal gyrus, SFG: superior frontal gyrus, Cing G: cingulate gyrus, ITG: inferior temporal gyrus, FFA: fusiform face area.
